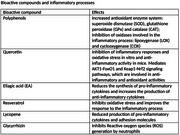# The neuroprotective action of bioactive compounds as a strategy for preventing mild cognitive decline

**DOI:** 10.1002/alz70855_102095

**Published:** 2025-12-23

**Authors:** Gustavo A A Santos, Mario RM Junior

**Affiliations:** ^1^ UNICAMP, Campinas, Brazil; ^2^ Sao Leopoldo Mandic Araras School of Medicine, Araras, Brazil; ^3^ USP ‐ University of Sao Paulo, Ribeirao Preto, Brazil

## Abstract

**Background:**

bioactive compounds found in berries are capable of reducing brain inflammatory activity, in addition to exerting neuroprotective activity, as they contain substances such as Quercetin, Ellagic Acid, among others, which reduce oxidative stress, inhibit beta amyloid aggregation, TAU hyperphosphorylation, regulating synaptic function, activating the Transcription Factor Nrf2.

**Method:**

This is a literature review carried out from August to December 2024, based on publications from the last 10 years that provided evidence regarding the neuroprotective results of bioactive compounds. The keywords used were: berries, TAU, Abeta, neuroprotection, anti‐inflammatory;

**Result:**

The secondary metabolites of bioactive compounds demonstrate the ability to prevent neuronal death as they are capable of reducing the levels of Reactive oxygen species, blocking the activation of caspase‐3; in addition to increasing redox activity.

**Conclusion:**

The use of bioactive compounds in the diet, in a regulated manner, or even as a supplement can be a protective factor to avoid mild cognitive decline and consequently dementia, such as Alzheimer's.